# The complete mitochondrial genome of *Glyptothorax cavia* (Siluriformes, Sisoridae, *Glyptothorax*): genome characterization and phylogenetic analysis

**DOI:** 10.1080/23802359.2017.1307701

**Published:** 2017-05-08

**Authors:** Bo Li

**Affiliations:** a Chinese Sturgeon Research Institute, China Three Gorges Corporation, Yichang, Hubei, China;; b College of Fisheries, Huazhong Agricultural University, Wuhan, China

**Keywords:** *Glyptothorax cavia*, mitochondrial genome, control region, phylogenetic relationship

## Abstract

*Glyptothorax cavia*, a small-sized benthic fish, distributed in southwest China. In the present study, the complete mitochondrial genome of *G. cavia* was sequenced to be 16,529 bp in length, including 13 protein-coding genes, 2 ribosomal RNAs, 22 transfer RNAs, a control region, and the origin of the light strand replication. The overall nucleotide composition was 31.15% A, 25.86% T, 27.64% C, and 15.35% G, with an A + T bias of 57.01%. The gene composition and the structural arrangement of the *G. cavia* complete mtDNA were identical to most of the other vertebrates. This will provide a useful tool for understanding the genetic diversity, population structure, and conservation status of *G. cavia* in the future.

## Introduction

Mitochondrial DNA (mtDNA) gene order was proposed to be quite conserved within vertebrates based on the gene order of the initial genome sequence (Anderson et al. 1981; Bibb et al. 1981). *Glyptothorax cavia* (Siluriformes: Sisoridae) is an endemic fish species which mainly distributes in the Irrawaddy, Nujiang River and their tributaries in China. In recent years, the natural resource of this species has seriously declined, as a result of overharvesting, water contamination, and especially dam construction (Shao et al. 2016). In the long run, a good understanding of the genetic diversity and population structure of *G. cavia* is required in order to establish adequate management plans for the conservation of this species. To address these topics, we determined the complete mitochondrial genome sequence of *G. cavia* for the first time.

Specimens of *G. cavia* were collected from Nujiang River (25°50′0.34″N. 98°51′33.76″E) in March 2015. The caudal fins of 12 specimens were cut-off and preserved in 95% ethanol, then stored under −80 °C until DNA extraction. Total genomic DNA was isolated from the caudal fin by proteinase K digestion followed by the standard phenol/chloroform method (Sambrook & Russell 2001) and visualized on 1.5% agarose gel. 20 sets of primers were designed for PCR amplification on the basis of aligned mitogenome sequences of *Glyptothorax trilineatus* with the Accession NC_021608.1. In order to avoid errors of assembly, the complete mtDNA genome was aligned and checked with three reported mtDNA genome sequences of Sisoridae species *G. zainaensis* (Accession NC_029709.1); *G. fokiensis* (Accession NC_018769.1); *G. trilineatus* (Accession NC_021608.1). The assembled sequence was analyzed using the software MitoAnnotator (Iwasaki et al. 2013) and nucleotide composition was calculated by MEGA6 (Tamura et al. 2013).

The complete mtDNA sequence of *G. cavia* reported here has been deposited in GenBank under the accession number KY230517. The mitochondrial genome of *G. cavia* is a circular molecule of 16,529 nucleotides, which is similar to other vertebrates, including 13 protein-coding genes, 2 ribosomal RNA genes, 22 transfer RNA genes, and a non-coding control region (D-loop). The overall nucleotide composition is 31.15%, 25.86%, 27.64%, and 15.35% for A, T, C, and G, with an A + T content of 57.01%, respectively. Except for a single protein-coding gene (*ND6*) and eight tRNA genes (*tRNA^Gln^*, *tRNA^Ala^*, *tRNA^Asn^*, *tRNA^Cys^*, *tRNA^Tyr^*, *tRNA^Ser^* (UCN), *tRNA^Glu^*, and *tRNA^Pr^°*) encoded on the L-strand. All the other genes were encoded on the H-strand. The first non-coding region is 885bp between *tRNA^Pr^°* and *tRNA^Phe^*, and the second one is the origin of light-strand replication, which extends up to 31bp. It is located in a cluster of five tRNA genes (the WANCY region) between *tRNA^Asn^* and *tRNA^Cys^* gene.

Furthermore, the termination codon varies with TAA, TA–, T––, or TAG. Virtually, all of the 13 protein-coding genes show the regular initiation codon ATG with the sole exception of *COI* which started with GTG. Six protein-coding genes terminated with the complete stop codon TAA (*ND1*, *COI*, *ATPase8*, *ND4L*, and *ND5*) or TAG (*ND6*), while the rest ended with incomplete stop codon T–– (*ND2*, *COII*, *COIII*, *ND3*, *ND4*, and *Cytb*) or TA– (*ATPase 6*), which is quite typical among mtDNA genes in other fishes (Zhou et al. 2012; Wang et al. 2013).

**Figure 1.**
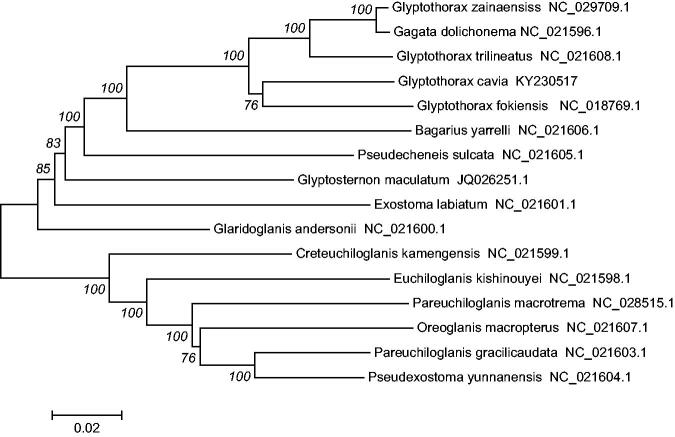
The consensus phylogenetic relationship of the *G. cavia* with other Sisoridae species. *O. macropterus, P. yunnanensis, P. gracilicaudata*, and *P. macrotrema* were used as an outgroup. The numbers along the branches are Bayesian posterior probability and bootstrap values for NJ, estimated for concatenated mitochondrial protein sequences.

In addition, the mtDNA sequences of 15 species of fishes were downloaded from Gen Bank, *Oreoglanis macropterus* (Accession NC_021607.1), *Pseudexostoma yunnanensis* (Accession NC_021604.1), *Pareuchiloglanis gracilicaudata* (Accession NC_021603.1), *P. macrotrema* (Accession NC_028515.1) were used as an outgroup for phylogenetic analysis. Phylogenetic analyses were performed using the neighbour joining (NJ) in MEGA 6.0 (Kumar et al. 2008). The tree topologies based on complete mt DNA sequences in this study were identical and were statistically supported by high bootstrap and posterior probability values (Figure 1). The mitogenome data provided strong support that *G. Cavia* was clustered together with *Glyptothorax fokiensis* (Accession NC_018769.1), *G. trilineatus* (Accession NC_021608.1), *Gagata dolichonema* (Accession NC_021596.1) and *G. zainaensiss* (Accession NC_029709.1). The phylogenetic analyses yielded convincing evidence that the genera *Gagata* and *Glyptothorax* constituted a monophyletic group and then formed a sister group with all *Bagarius yarrelli* (Accession NC_021606.1).
